# The effectiveness of simulation-based learning (SBL) on students’ knowledge and skills in nursing programs: a systematic review

**DOI:** 10.1186/s12909-024-06080-z

**Published:** 2024-10-07

**Authors:** Ali Alharbi, Arina Nurfianti, Rosemary F. Mullen, John D. McClure, William H. Miller

**Affiliations:** 1 School of Medicine, Dentistry & Nursing, College of Medical, Veterinary & Life Sciences, University of Glasgow, Glasgow, Scotland; 2 College of Nursing, King Saud University, Riyadh, Saudi Arabia; 3 School of Nursing, Universitas Tanjungpura, Pontianak, Indonesia; 4 School of Cardiovascular and Metabolic Health, College of Medical, Veterinary & Life Sciences, University of Glasgow, Glasgow, Scotland

**Keywords:** Simulation-based learning, Manikin, Mannequin, Simulator, Knowledge, Skills, Nursing students

## Abstract

**Background:**

Simulation-Based Learning (SBL) serves as a valuable pedagogical approach in nursing education, encompassing varying levels of fidelity. While previous reviews have highlighted the potential effectiveness of SBL in enhancing nursing students’ competencies, a gap persists in the evidence-base addressing the long-term retention of these competencies. This systematic review aimed to evaluate the impact of SBL on nursing students’ knowledge and skill acquisition and retention.

**Method:**

A comprehensive search of electronic databases, including CINAHL, PubMed, Embase, Scopus, and Eric, was conducted from 2017 to 2023 to identify relevant studies. The Joanna Briggs critical appraisal tools were used to assess the methodological quality of the included studies. A total of 33 studies (15 RCTs and 18 quasi-experimental) met the inclusion criteria and were included in the review. A descriptive narrative synthesis method was used to extract relevant data.

**Results:**

The cumulative sample size of participants across the included studies was 3,670. Most of the studies focused on the impact of SBL on life-saving skills like cardiopulmonary resuscitation (CPR) or other life-support skills. The remaining studies examined the impact of SBL on critical care skills or clinical decision-making skills. The analysis highlighted consistent and significant improvements in knowledge and skills. However, the evidence base had several limitations, including the heterogeneity of study designs, risk of bias, and lack of long-term follow-up.

**Conclusion:**

This systematic review supports the use of SBL as a potent teaching strategy within nursing education and highlights the importance of the ongoing evaluation and refinement of this approach. While current evidence indicates enhancing knowledge and skill acquisition, limited studies evaluated the retention beyond five months, constraining generalisable claims regarding durability. Further research is essential to build on the current evidence and address gaps in knowledge related to the retention, optimal design, implementation, and evaluation of SBL interventions in nursing education.

**Supplementary Information:**

The online version contains supplementary material available at 10.1186/s12909-024-06080-z.

## Background

Simulation-Based Learning (SBL) is an educational approach which has been widely adopted in nursing and medical education [1]. The predominance of this approach can be understood to relate to the way in which SBL seeks to replicate aspects of real-world situations, allowing students to apply knowledge and develop their practical skills in a safe environment [2]. This is valuable for nursing, a field which relies on the practical application of skills [2]. The intended outcome of SBL could be to enhance the acquisition of knowledge and skills as well as the retention of these over time [3]. These outcomes demonstrate the immediate effectiveness of SBL and its long-term impact on students’ competence. Despite its widespread use, there is a lack of evaluation of the efficacy of SBL within nursing education in achieving immediate and long-term knowledge and skills. Therefore, this review aims to evaluate how SBL impacts knowledge and skills among nursing students.

Simulation training has demonstrated substantial value in developing nurses’ resuscitation and critical care abilities. Performing high-quality cardiopulmonary resuscitation (CPR), responding to patient deterioration events, and managing crisis situations require sophisticated psychomotor and clinical judgment proficiencies [4]. Additionally, critical care environments involve complex technologies and rare emergency scenarios that learners may inconsistently encounter through conventional clinical education alone [5]. Thus, simulation-based mastery learning has emerged as an efficacious approach for standardising novice nurses’ exposure to low-frequency, high-risk contexts requiring rapid emergency response capabilities and proficient use of specialised equipment.

SBL can be delivered via several modalities: high, medium, and low fidelity [6, 7]. High-fidelity SBL sessions seek to recreate a patient scenario with a high degree of realism [8], whereas, in contrast to this, low-fidelity SBL still focuses on practising the target skills, but in an environment which was less reminiscent of the dynamics or pressures of real-world practice [9]. Therefore, high-fidelity SBL might be expected to be more educationally valuable than low-fidelity SBL, however, the evidence does not entirely support this supposition. For example, a recent study conducted by Massoth et al. [10]. compared high, and low-fidelity SBL approaches for an advanced life support training session. Their findings indicated that improvements in knowledge and skills for those who experienced high-fidelity SBL were not significantly different to those who had undertaken low-fidelity SBL [10]. Furthermore, Massoth et al. sub-item analysis indicated that high-fidelity SBL participants were prone to becoming overconfident with the given task, which Massoth et al. view as an undesirable side effect of such an approach.

It is, therefore, important to examine SBL in more detail through a thorough a review of the literature. This will address the question of whether SBL meets its objectives for knowledge and skills acquisition and retention and may also help resolve ongoing debates relating to fidelity. Recent reviews have undertaken important preparatory work in examining this area: the integrative review of Al Gharibi and Arulappan [11] evaluated SBL on a range of outcomes for nursing students, as did the systematic review conducted by Labrague et al. [12]. However, both reviews focussed on core outcomes relating primarily to the confidence of learners and did not specifically examine issues of knowledge and skills acquisition or retention.

This systematic review aims to critically appraise and synthesize the published evidence on the effectiveness of SBL on students’ knowledge and skills acquisition and retention in nursing programs.

## Methods

The protocol for this systematic review was developed and registered on PROSPERO, the registration number CRD42021284544 and was reported in accordance with the Preferred Reporting Items for Systematic Reviews and Meta-Analyses (PRISMA) quality requirements [13].

### Search strategy

The article selection entailed two phases: initial scoping and a strategic search [14]. The systematic search utilized five leading academic databases: CINAHL, PubMed, Embase, Scopus, and Eric, employing the Population, Intervention, Comparison, and Outcomes (PICO) framework to establish precise inclusion criteria [14, 15]. Only English-language studies were included, with key terms such as simulation-based learning, education, training, and related synonyms. The terms were merged using Boolean operators and tailored for each database as needed. The databases indexed all major relevant journals, eliminating the need for manual searches. Reference lists in the review were examined for further sources. All chosen articles were published within seven years of this study, aligning with the field’s rapidly evolving nature and ensuring the incorporation of recent advancements [16]. This timeframe strikes a balance between recency and a sufficient depth of literature, offering a comprehensive overview of current trends and methodologies (Table 1).

**Table 1:** Literature search strategy

Search Items	Simulation-Based learning; manikin mannequin, simulator; advanced cardiac life support; basic cardiac life support Knowledge Skills Nursing students.
Databases Searched	CINAHL, PubMed, Embase, Scopus, and Eric
Parts of Journals Searched	Titles, abstracts, and body text
Date Range	2017–2023
Language	English
Research Design	Randomized controlled trials Pre- and post-test design Quasi-experimental design
Inclusion Criteria	Nursing students (undergraduate/ postgraduate) Knowledge, skills acquisition and retention.
Exclusion Criteria	Unavailable in English Incorrect population Conference paper

### Screening process

References obtained from the database search were organized and imported via EndNote X9 reference management software. Covidence systematic review software was employed to streamline the screening and selection procedures [17]. Two reviewers, AA and AN, independently examined the abstracts, titles, and full texts of all records to ascertain eligibility based on inclusion criteria. A third reviewer (RM) was consulted for consensus in cases of discrepancies regarding study eligibility.

### Data extraction

Upon securing the final articles, an extraction form was devised and pilot-tested to abstract salient study characteristics and outcomes [18], in compliance with rigorous guidelines for systematic reviews [19]. Data pertinent to the PICO framework and encompassing both cognitive and psychomotor domains were extracted. Reviewers AA and AN evaluated the form’s feasibility. To ensure data integrity and mitigate bias, AA and AN undertook the data extraction, which was subsequently corroborated by an additional pair of reviewers (RM and WM). The characteristics of the studies incorporated in this review are succinctly encapsulated in the supplementary file, which delineates authorship, publication year, geographic origin, objectives, methodology, participant demographics, simulation activities, and key findings germane to the review.

### Assessment of the risk of bias in included studies

Reviewers AA and AN independently scrutinized full-text articles utilizing the Joanna Briggs Institute’s (JBI) critical appraisal tools [20]. JBI, an independent, international, non-profit research entity affiliated with the University of Adelaide’s Health and Medical Sciences faculty, has devised an array of critical appraisal checklists to assess healthcare interventions’ feasibility, appropriateness, meaningfulness, and efficacy [21]. The JBI checklist for Randomised Controlled Trials and Quasi-Experimental Studies was selected for their relevance to the study designs targeted in this review, encompassing thirteen and nine items respectively, addressing aspects such as design, sample selection, and comparison. Items are scored dichotomously, with a maximum aggregate score of 13 for RCTs and 9 for quasi-experimental studies, facilitating a holistic assessment of each study’s quality. Further, methodological judgment will also be incorporated into the quality assessment.

Irrespective of the methodological quality, all selected studies were integrated into the review. This approach was adopted to ensure a comprehensive synthesis of the available evidence on SBL concerning knowledge and skill in nursing education. Excluding studies based on methodological quality alone might omit potentially valuable insights. Including a range of studies allows for an understanding of the current evidence base and highlights areas needing further methodological refinement. This inclusive strategy enables a holistic view of the research landscape [22]. Reviewers AA and AN independently performed the quality assessment, with discrepancies adjudicated by a third reviewer (RM). Quality scores were tabulated utilizing a spreadsheet template in Microsoft Excel, deploying a categorical response set (“yes”, “no”, “can’t tell”, or “not applicable”).

### Data synthesis strategy

This review adopted a descriptive narrative synthesis approach [23], which systematically outlines and synthesises key characteristics and evidence across the selected studies, offering a comprehensive summary of the findings. All the included studies applied simulation to a range of clinical topics using a variety of methods, but similar outcomes (knowledge and skill acquisition and retention) and interventions were used. This involved the discussion and reporting of critical and comparative details about the simulation interventions as well as the characteristics of the focus population, the types of outcomes measured and the overall quality of the study. The narrative synthesis also included a textual description of the simulation intervention methodology. The approach allows for articulating the congruities and disparities among the studies concerning methodological quality, design, methodology, outcome measures, and findings [24].

## Results

### Study selection

**Fig. 1:**
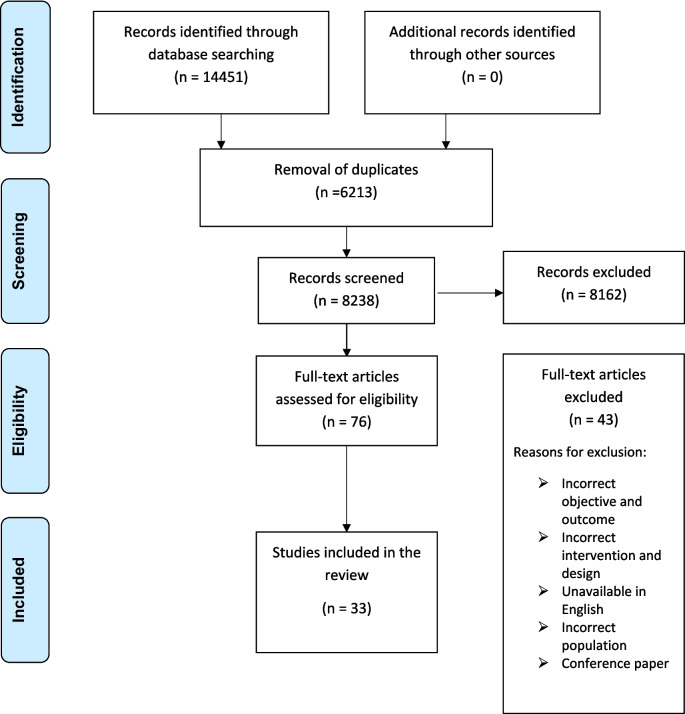
PRISMA Flow Diagram

The scientific database returned 14,451 articles, of which 6,213 duplicates were excluded. Titles and abstracts of the remaining 8,238 articles were assessed for eligibility using Covidence systematic review software. This initial screening excluded 8,162 articles due to irrelevance. Subsequently, 76 articles underwent full-text screening; 33 met the inclusion criteria and were further assessed by AA and AN, while 29 were excluded. The PRISMA flow diagram [25] in Fig. 1 summarizes the search and selection process.

### Study characteristics

A total of 33 papers, published between 2017 and 2023, met the inclusion criteria (Table 2). Table 3 delineates the studies’ attributes, encompassing research design, simulation interventions, and result of appraisal & quality rating. These studies exclusively assessed SBL in augmenting nursing students’ knowledge and skills. Among them, 31 utilized a quantitative approach, while Demirtas et al. [26] and Zieber and Sedgewick [27] employed mixed methods; however, only quantitative results are discussed in this review.

Fifteen studies employed Randomised Controlled Trials (RCTs) [28–42]. While the remainder (*n* = 18) adopted quasi-experimental designs [26, 27, 43–58].

The studies were globally distributed, comprising eight from Europe, two from South America, one from Australia, four from North America, seven from the Middle East, and eleven from Asia, collectively, these encompassed 19 countries.

All 33 studies focused on student nurses, resulting in an aggregated sample size of 3,670 participants. Sample sizes varied considerably, ranging from 16 of Hardenberg et al. [30] to 479 Requena-Mullor et al. [51]. Notably, nine studies had over 100 participants. One study of Hardenberg et al. [30] exclusively involved postgraduate students, while the remainder encompassed a range of undergraduate levels. Although Ka Ling et al. [33] and Kardong-Edgren et al. [50] recruited from multiple nursing schools, the others were confined to single institutions.

Simulation fidelity varied across studies which creates difficulties when comparing and synthesizing results between simulations that are not standardised. For example, when different topics are taught to different groups of participants, different time allocations are made, different roles are played, a variety of data collection tools are employed, and different levels of fidelity are used.

Regarding the SBL interventions, a majority (*n* = 23) examined cardio-pulmonary resuscitation (CPR) or other life support skills [26, 27, 29, 31–34, 36, 37, 40–45, 47–51, 53, 55, 56]. The rest focus on critical care skills [30, 35, 38, 39, 46, 52, 54, 58] or clinical decision-making skills [28].

**Table 2:** Characteristics of included studies

Primary author (Year)	Aim	Study design	Participants (sample size)	Simulation (intervention)	Results	finding Related to the review purpose
Araújo, Medeiros [34] Brazil	To identify the effect of clinical simulation on the immediate and retained cognitive performance of nursing students in a vocational course about their performance in emergencies.	RCT with repetitive test measure	46 nursing students	Practice in the scenario of experiencing a situation of cardiopulmonary arrest in adults, pregnant women, and a choking situation.	Students in the experimental group retained more knowledge than those in the control group.	Students who learned from the simulation methodology retained better cognitive performance than a traditional teaching strategy.
Arrogante, Rios-Diaz [29] Spain	To analyse the effects of deliberate practice using a feedback device (FD) on the CPR performance of nursing students before, immediately after, and three months after training.	RCT	60 nursing students	Structured CPR training.	The participants in the training group had an improved ability to perform chest compressions and ventilations and improve their overall quality of CPR than the control group.	Simulation has a positive effect on the acquisition of CPR skills by participants.
Charlier, Van Der Stock [43] Belgium	- To identify which basic life support skills of student nurses deteriorate in a period of four months. - To investigates the link between a specific cognitive skill and its corresponding motor skill in BLS.	Quasi-experimental repeated measure	169 nurse students	A standardised BLS-AED face-to-face course.	On BLS knowledge, 65% of students passed the overall post-test. 61% of all students passed the retention test. BLS skills were significant in skill performance between post and retention.	SBL was an effective method for teaching BLS knowledge and skills. There was a significant correlation between knowledge and skills in their acquisition and retention.
Chen, Yang [48] China	To evaluate the impact of a standardised simulation-based emergency and intensive care nursing curriculum on nursing students’ response time in a resuscitation simulation	Two-group, non-randomised quasi-experimental design.	39 third-year nursing students	Participated in standardised simulation-based in the following: clinical priorities (e.g. triage), basic resuscitation skills, airway/breathing management, circulation management and teamwork.	There was a significant decrease in the median ± IQR in seconds elapsed between the call for assistance and the initiation of chest compressions in the experimental group. Similar decrease from the call for help to successful defibrillation.	SBL methodology was associated with decreased response time in a resuscitation.
D’Cunha, Fernandes [44] India	To evaluate the effectiveness of a simulation-based training program for nursing students involved in simulated Code Blue resuscitation scenarios.	Interventional study, Pre and post	65 Nursing students	Hands-on stations on the crash cart and cardiac arrest algorithms using an electrocardiogram simulator.	A significant increase in mean% from pre-test to post-test (55.69–77.33%) following simulated drills.	Using simulation to train nurse students in Code Blue scenarios records greater satisfaction and improvement in clinical reasoning, knowledge, and skills.
Demirtas, Guvenc [26] Turkey	To examine the effectiveness of a simulation-based CPR training program on the knowledge, practices, satisfaction, and self-confidence of nursing students.	mixed-method design, with Pre and post-design (for quantitative).	89 fourth-year nursing students	Theoretical training for 45 min, then demonstrated CPR on a medium-fidelity CPR model according to the 2015 AHA guideline. 15 min performed CPR skills in the scenario.	The mean score of CPR knowledge score increased significantly after the simulation (*p* < 0.001). The mean post-test CPR skills score was significantly higher than the mean pre-test CPR skills score (*p* < 0.001).	SBL improved the level of knowledge and skills of nursing students. In addition SBL had a positive effect on students’ satisfaction and self-confidence.
Farsi, Yazdani [42] Iran	To evaluate the difference in educating nursing students on CPR when using the traditional simulation training with a mannequin versus a more novel serious game training on the smartphone platform.	RCT	56 Nurse students	A simulation-based CPR training, CPR training using a serious game on the smartphone platform, and a control group that received no CPR training.	The simulation and serious game intervention groups demonstrated better scores on the knowledge questionnaire and on the CPR skill demonstration.	CPR training for nursing students using simulation training and the novel serious game training on a smartphone platform both increased CPR knowledge and skill.
Filomeno, Renzi [45] Spain	To evaluate the effectiveness of SBL method, as an additional method to theoretical lectures, on improving critical care knowledge.	A non-experimental pre-test and post-test study	60 nursing students	Participating in four scenarios using simulation: ABCDE assessment, disturbance identification, prioritization and application of algorithms	Significance improvement of the intervention in the post-test: p-value 0.01 and the students improved on average by 1 point after the intervention, passing from 11.94 in the pre-test to 12.94 in the post-test.	Nursing students involved in the critical care course needed the simulated clinical scenarios to improve their abilities to solve questions or problems related to critical care.
Goldsworthy, Patterson [46] Canada	To evaluate the effects of SBL on nursing students’ confidence and competence in the recognition and response to rapidly deteriorating adult and paediatric patients.	Quasi-experimental design	63 undergraduates nursing students in final year	Participated in High-fidelity cases in six scenarios (angina/cardiac arrest, COPD/respiratory failure, post-op haemorrhage, paediatric sepsis, paediatric asthma, neonatal seizures)	Significant improvement in all items on the Clinical Self-efficacy tool was seen in the treatment group after the intervention. On the contrary, there was no significant improvement in any of the Clinical Self-efficacy items in the control group.	Simulation effective in improving confidence and competence in the recognition and response to deteriorating patients. Further, significant increases in knowledge (myocardial infarction, paediatric asthma, and septic shock).
Habibli, Ghezeljeh [31] Iran	To investigate the effect of SBL on the knowledge and performance of nursing students of adult essential life support cardiopulmonary resuscitation (BLS-CPR).	RCT	49 nursing students	The participants practice various BLS roles for 5 min, including heart compression, artificial respiration, and the use of AED.	The mean scores of students’ knowledge and performance in the intervention group immediately after (*p* < 0.001) and three months after the intervention (*p* < 0.05) were significantly higher than the control group.	SBL increased the knowledge and performance of nursing students in the field of BLS-CPR. Integrating conventional training with simulation-based education can be effective in learning BLS among nursing students.
Hardenberg, Rana [30] Australia	To assess the impact of HFS training on four vital skill areas: focused patient assessment, primary response, consultation with the doctor and emergency management interventions in postgraduate critical care nursing students	RCT	16 post-graduate nursing (Master of Nursing).	a 90-minute training in two stations. The first station with a human simulation manikin to resemble a critically ill patient. The second is the training simulation on airway management.	Focused patient assessment and emergency management intervention skills were improved and higher on training simulation group compared to the control group (*p* < 0.05).	HFS can impact the skills area of ‘‘focused patient assessment’’ and ‘‘emergency management intervention.’’
Ka Ling, Lim Binti Abdullah [33] Malaysia	To compare learning outcomes using an adult code blue resuscitation drill simulation, by using high fidelity patient simulation (HFPS) versus low fidelity patient manikins (LFPM).	RCT, pre-test and post-test double-arm intervention study.	409 third-year nursing students.	Participating in a code blue scenario to assess participants’ knowledge of CPR, administration of medication, identification of life-threatening arrhythmias, and team collaboration on a deteriorating patient.	A repeated-measures ANCOVA showed significant differences in knowledge levels and CT skills between control and intervention groups. The intervention group showed a significant improvement compared to control group.	Simulation-based education using HFPS can help increase knowledge and CT skills among nursing students in code blue management.
Kardong-Edgren, Oermann [50] USA	To compare the CPR psychomotor skills nursing students to assess their retention of skills post-BLS certification and following one training session using the RQI system.	Pre and post-design.	467 nursing students	Students initially completed 60 s of chest compressions and 1 min of bag, valve, and mask ventilation.	Overall compression and ventilation scores improved scores improved significantly from pre-test to post-test *p* < 0.0001 increase following a 10-minute RQI coached session.	SBL help Students to improve their skills in compression and ventilation.
Keys, Luctkar-Flude [32] Canada	To evaluate the effect of a resuscitation-oriented VSG (Virtual simulation gaming), when implemented as a pre simulation preparation adjunct, on the performance of nursing students during an advanced cardiac life support.	a parallel group randomised controlled trial (RCT) with a 1:1 intervention to control allocation ratio	26 nursing students	Each learner was required to complete one V-Fib arrest simulation, which focused on assessment and team roles during resuscitation. For participants allocated to the intervention group, the VSG is a 15-minute computer-based game that allows users to experience the role of a nurse caring for a patient in cardiac arrest.	Overall performance was greater for participants in the in- intervention group (mean = 12) than for the control group (mean = 8).	Virtual simulation gaming could serve as an effective pre simulation preparation tool
Kim, Issenberg [37] Korea	To identify the effects of simulation-based advanced life support education on nursing students’ knowledge, performance, self-efficacy, and teamwork.	RCT	60 fourth-year nursing students	Practised airway management and CPR (bag-valve-mask ventilation, chest compression, drug injection, and defibrillation), which were required in the high-fidelity simulation session.	The experimental group showed a higher mean score of knowledge, performance, and self-efficacy scores compared to the control group. There was no significant mean difference in the overall teamwork score.	SBL had positive effects on knowledge, performance, and self-efficacy among participants.
Lau, Chee [53] Singapore	To evaluate the team performance of students after Interprofessional simulation-based advanced cardiac life support (IPSACLS) training	Pre and post-design.	80 nursing and medical students	The simulation experience comprised nine different simulation scenarios, and each group needed to finish nine stations in two days. Simulation sessions involved using a high-fidelity simulation manikin in a team approach.	The total scores of the Clinical Teamwork Scale and Communication and Team Skills Assessment were improved substantially after the IPS-ACLS (*p* < 0.001).	IPS-ACLS training could augment teamwork performance among nursing and medical students.
Li, Lv [40] China	To explore the impact of integrating online virtual simulation with interactive exercises and offline low fidelity simulation on CPR (cardiopulmonary resuscitation) skills of first-year nursing students.	RCT	72 first-year nursing students	Teaching cardiopulmonary resuscitation (CPR). Blended learning approach integrating virtual simulation with interactive exercises and offline low-fidelity simulation	The experimental group exhibited significantly greater improvement in their self-directed learning (SDL) abilities and significantly better performance in their CPR skills. There were no statistical differences in terms of their critical thinking abilities.	The simulation was effective in improving the students’ CPR skills and self-directed learning abilities
Meneghesso, Marcatto [55] Brazil	To verify the contributions of using the “blindfolded” technique on nursing students’ self-confidence and knowledge in critical patient care in simulated clinical scenarios.	Quasi-experimental design	25 Nursing students	Blindfolded leader to the simulated ACLS scenario using High fidelity manikin	There was a mean increase of 4.04 correct answers in the sample when compared to their baseline knowledge. The students in leadership roles exhibited a significant rise in their self-confidence during the care provided in critical scenarios.	Using the “blindfolded” simulation technique led to a significant increase in nursing students’ knowledge.
Padilha, Machado [35] Portugal	To evaluate the effect of clinical virtual simulation with regard to knowledge retention, clinical reasoning, self-efficacy, and satisfaction with the learning experience among nursing students.	RCT with repetitive test measure.	42 nursing students in the second year.	A 45 min of class in the field of the respiratory process about ineffective airway clearance and hypoxia with recourse to a clinical virtual simulator scenario	The experimental group had better outcomes in knowledge after the intervention (*P* = 0.001), retention two months later (*P* = 0.02) and learning satisfaction (*P* < 0.001) than the control group.	SBL improves knowledge retention and clinical reasoning over time and improves student satisfaction with learning.
Requena-Mullor, Alarcón-Rodríguez [51] Spain	To evaluate the effects of the BLS clinical simulation course on Undergraduate Nursing Students’ Learning.	A pre-post intervention study.	479 nursing students.	30 min of BLS simulation session, including and proper use of the automated external defibrillator (AED).	statistically significant differences in the total score of the pre-test and after completing the BLS course (pre-test (12.61), post-test (15.68), *p* < 0.001). A significant increase in the mean scores was observed in each of the four parts of the assessment protocol (*p* < 0.001).	SBL was an effective method of teaching and learning BLS skills, and it is recommended that nursing students repeat BLS training throughout their education.
Roh, Kim [54] Korea	To identify the effects of a nursing simulation program with team-based learning (TBL) on Korean nursing students’ knowledge, team performance, and teamwork.	A one-group pre and post-test design	229 fourth-year nursing students.	Participate in three patient scenarios (hyperglycaemia, breathing difficulty, and cardiac arrest) using the human patient simulator. The fourth session for communication skills was a handover simulation performed by the students in pairs using scenarios that had been conducted previously.	The median post-test rank on team performance was significantly higher than the median pre-test rank (z = − 10.09, *P* < 0.001). Participants achieved higher scores in the Group Readiness Assurance Test than they did in the Individual Test	A simulation program positively affected nursing students’ knowledge, performance, and teamwork.
Saeidi and Gholami [36] Iran	To assess the effect of SBE on nursing students’ knowledge	RCT	80 s year nursing students	A 5-hour neonatal resuscitation session, according to NRP 2016 was used for the SBE group.	The pre-test analysis did not show any significant differences in knowledge between the two groups, but the post-test analysis showed that the SBE group had significantly higher knowledge.	SBL was significantly more effective than traditional in the students’ knowledge of neonatal resuscitation education.
Sapiano, Sammut [52] Malta	To investigate the effectiveness of virtual simulation in improving student nurses’ knowledge and performance during rapid patient deterioration.	A pre- and post-test design.	166 undergraduate nursing students.	The students took part in three virtual scenarios in which they had to assess and manage rapid patient deterioration. They had to complete each scenario within 8 min, prompting them to act quickly and prioritise care appropriately.	The mean pre-scenario knowledge scores were 6.71 out of 11, the mean post-scenario knowledge scores were 7.61, indicating an improvement in the students’ knowledge in cardiac, shock respiratory scenario.	Virtual simulation programs may improve knowledge and improve performance in deterioration management.
Seo and Eom [39] Korea	To assess the effect of a simulation nursing education program on clinical reasoning, problem-solving process, self-efficacy, and clinical competency using the Outcome-Present State-Test (OPT) model in nursing students.	RCT	45 nursing students.	The simulation session comprised gastrointestinal tract bleeding and acute myocardial infarction to reflect the opt model.	A significant improvement in clinical reasoning (*p* = 0.002), problem-solving process (*p* < 0.001), and self-efficacy (*p* < 0.001) and clinical competency in the experimental group as compared to the control group.	SBL effectively improved clinical reasoning, problem-solving process, self-efficacy, and clinical competency in undergraduate nursing students.
Seol and Lee [49] Korea	To identify the effects of CPR training among nursing students in Mozambique.	A one-group pre-test and post-test repeated-measures quasi-experimental design.	32 nursing students.	Practical training for CPR was conducted for 2 h using two CPR manikins (Little Anne, Laderal^®^).	Attitude and self-efficacy scores of students on CPR significantly increased immediately after CPR training but decreased 20 weeks after the intervention.	SBL positively affected attitude and self-efficacy in CPR among RN-BSN nursing students immediately, but not at 20 weeks, after the training.
Svellingen, Forstrønen [28] Norway	To assess the effect of multiple simulations on the students’ self-reported clinical decision-making skills and self-confidence.	RCT with two arms (double scenario simulations as intervention and single scenario simulations as control) and three follow-up time points.	146 nursing students	One day of simulation during the first academic year, two simulation days the second year, and one during the third year.	No significant differences between double vs. single scenario sessions on clinical decision-making scores or self-confidence scores.	The overall self-confidence scores increased significantly over time after simulation intervention.
Sarvan and Efe [41] Turkey	To determine the impact of integrating serious game simulation (SGS) into neonatal resuscitation training on the neonatal resuscitation related knowledge, skills, satisfaction with training, and self-confidence in learning of nursing students.	RCT with pre and post-test measure.	90 nurse students.	A neonatal resuscitation algorithm and employed a neonatal resuscitation serious game simulation (SGS) method to allow students to engage in neonatal resuscitation scenarios in a simulated, game-based environment.	The intervention group showed significantly better ventilation and chest compression skills, with p-values of 0.011 and 0.020, respectively. Both groups demonstrated a significant increase in neonatal resuscitation knowledge and skills post-training (*p* < 0.05), and High scores in satisfaction and self-confidence	The serious game simulation method used in neonatal resuscitation training effectively enhanced the students’ ventilation and compression performing skills.
Tucker, Urwin [56] UK	To investigate the impact of unsuccessful resuscitation and manikin death during simulation on nursing student’s resuscitation self-efficacy.	A quasi-experimental design.	120 s year nursing student	The simulation session was split into two groups, with one group being assigned to the scenario where the patient was successfully resuscitated and the other group going into the unsuccessful resuscitation scenario.	Overall, both groups showed improved self-efficacy because of the simulation session and the death of the manikin in the experimental group did not result in a reduced level of self-efficacy related to resuscitation	Skills and AED used significantly improved between pre- and post-simulation.
Tawalbeh [38] Jordan	To examine the effects of simulation modules on students’ knowledge and confidence in performing critical care skills.	RCT with repetitive test measure.	76 nurse students.	Three scenarios were implemented for each system and 18 h of simulation were provided for each group in the experimental group. A high-fidelity simulator was used with features including ventilation, hemodynamic monitoring, electrocardiogram analysis, medication administration, and chest tube.	A statistically significant difference between the control and experimental, the students in the experimental group scored significantly higher *p* < 0.001 than the control group in both knowledge and confidence regarding performing critical care skills.	Simulation significantly impacts nursing students’ knowledge and confidence in implementing critical care nursing skills.
Tseng, Hou [58] Taiwan	To determine the impact of combining clinical simulation scenario training and Information Technology Integrated Instruction (ITII) on the teaching of nursing skills.	A quasi-experimental design.	120 fourth year nursing student	Clinical scenario based on five cardiac topics: (PTCA) and (AMI); (BLS) and (AED) (SDH) care; applications Simulator for patients with septic shock; and acute respiratory distress syndrome (ARDS) care.	Experiment group significantly better OSCE performance, lab scores and improvements from the previous year’s grades.	SBL showed better in summative evaluation of knowledge components, OSCE formative evaluation and clinical nursing internship scores.
Tuzer, Inkaya [47] Turkey	To compare the effect of training with high-fidelity and medium-fidelity simulator cardiopulmonary resuscitation manikins on nursing students’ knowledge and performances.	Quasi-experimental design.	90 third-year nursing students who completed the “First Aid and Emergency Care”	Perform CPR on a Medium (experimental group 2) and high (experimental group 1) Fidelity CPR manikin.	Knowledge scores in both groups demonstrated significant increases in scores. According to log reports of the Medium Fidelity CPR manikin, correct compression rate and wrong hand position rate were found to change significantly over time in both groups.	Both HFS and MFS groups increased their level of CPR knowledge after training.
Yang and Oh [57] South Korea	To examine the effects (neonatal resuscitation nursing knowledge, problem-solving and clinical reasoning ability, self-confidence in practical performance, degree of anxiety, and learning motivation) using virtual reality.	A quasi-experimental study with a pretest-post-test design.	83 nursing students from two universities in South Korea	Virtual Reality Group received neonatal resuscitation gamification program using virtual reality. Simulation Group received high-fidelity neonatal resuscitation simulations in addition to online neonatal resuscitation program lectures.	The virtual reality and simulation groups had significantly higher scores in neonatal resuscitation knowledge and learning motivation scores compared to the control group. The virtual reality group show a significantly higher problem-solving ability, Self-confidence and exhibited significantly lower levels of anxiety compared to both the simulation and control groups.	The neonatal resuscitation gamification program using immersive virtual reality was effective in improving neonatal resuscitation knowledge, problem-solving ability, self-confidence, and learning motivation among nursing students.
Zieber and Sedgewick [27] Canada	To examines the relationship between competence, confidence, and knowledge retention in undergraduate nursing students.	A mixed quantitative and qualitative approach. The quantitative component comprised a repetitive test design.	24 students in the third or fourth undergraduate nursing.	One-day seminar similar in content to the AHA, Advanced Cardiac Life Support (ACLS) program. A three-hour knowledge session followed by a three hour high fidelity simulation activity.	The intervention was effective in improving perceptions of competence and confidence both immediately and at a three-month timeframe. Knowledge retention also was statistically significant at a three-month timeframe.	SBL effectively impact in confidence, competence, and knowledge both immediately and at a three-month interval.

### Methodological quality of the included studies

The appraisal outcomes are detailed in Appendix A & B. Following Reilly et al. [59]and Munn et al. [60], the overall quality was classified based on the proportion of criteria met (< 50% as poor, 50–80% as moderate, > 80% as good) (See Table 3). Discrepancies between reviewers were reconciled through team discussions.

### Risk of bias in studies

The studies were evaluated across six domains: selection bias, performance bias, detection bias, attrition bias, reporting bias, and other biases as per Higgins et al. [61], utilizing the JBI appraisal tool [20] for RCT and quasi-experimental studies. Measures taken to mitigate bias are reported in line with the criteria by Omura et al. [62] and Mbuzi et al. [63].

Among the 15 RCTs, all of the relevant studies ensured that all treatment groups were similar at a baseline level (selection bias), they addressed the potential risk of bias through randomisation. Only five studies [35, 38, 40–42] reported the concealment of treatment allocation.

Given the educational nature of interventions, instructors and assessors were often not blinded. A blind design was employed in five RCTs, with three blinding instructors and outcome assessors [29, 30, 32], and two blinding participants, instructors, and assessors [33, 41]. All RCTs completed follow-ups and used appropriate statistical analyses based on consistent participant groups pre-and post-intervention.

Regarding the 18 quasi-experimental studies devoid of random allocation, five consisted of pre-and post-intervention studies with a control group [46, 48, 56–58]. Overall, the 18 quasi-experimental studies employed identical measures on the same participants, before and after exposure to the intervention, follow-up was completed, and appropriate statistical analyses were conducted. However, these designs were not randomised and, therefore, they could contribute to potential bias. See Appendix A and B in the additional files for the full quality assessment table and the risk of bias judgements.

According to the JBI Evidence criteria [22], evidence from 15 RCTs was rated Level 1 C, while the 18 quasi-experimental studies was rated Level 2 C.

**Table 3:** Summary of characteristics of included studies and the result of the appraisal

Author (year) Country	Design	Simulation intervention	Result of appraisal &quality rating
Araújo, Medeiros [34] Brazil	RCT	BLS	9/13 (69%) Moderate
Arrogante, Rios-Diaz [29] Spain	RCT	BLS	10/13 (77%) Moderate
Farsi, Yazdani [42] Iran	RCT	BLS	9/13 (69%) Moderate
Habibli, Ghezeljeh [31] Iran	RCT	BLS	8/13 (62%) Moderate
Hardenberg, Rana [30] Australia	RCT	Critical care	10/13 (77%) Moderate
Ka Ling, Lim Binti Abdullah [33] Malaysia	RCT	ACLS	11/13 (85%) Good
Keys, Luctkar-Flude [32] Canada	RCT	ACLS	10/13 (77%) Moderate
Kim, Issenberg [37] Korea	RCT	ACLS	8/13 (62%) Moderate
Li, Lv [40] China	RCT	ACLS	10/13 (77%) Moderate
Padilha, Machado [35] Portugal	RCT	Critical care	10/13 (77%) Moderate
Saeidi and Gholami [36] Iran	RCT	NRP	7/10 (54%) Moderate
Sarvan and Efe [41] Turkey	RCT	NRP	11/13 (85%) Good
Seo and Eom [39] Korea	RCT	Critical care	9/13 (69%) Moderate
Svellingen, Forstrønen [28] Norway	RCT	Critical care	8/13 (62%) Moderate
Tawalbeh [38] Jordan	RCT	Critical care	10/13 (77%) Moderate
Charlier, Van Der Stock [43] Belgium	Quasi-experimental	BLS	8/9 (88%) Good
Chen, Yang [48] China	Quasi-experimental	BLS	8/9 (88%) Good
D’Cunha, Fernandes [44] India	Quasi-experimental	ACLS	8/9 (88%) Good
Demirtas, Guvenc [26] Turkey	Quasi-experimental	BLS	8/9 (88%) Good
Filomeno, Renzi [45] Spain	Quasi-experimental	Critical care	7/9 (78%) Moderate
Goldsworthy, Patterson [46] Canada	Quasi-experimental	Critical care	8/9 (88%) Good
Kardong-Edgren, Oermann [50] USA	Quasi-experimental	BLS	5/9 (56%) Moderate
Lau, Chee [53] Singapore	Quasi-experimental	ACLS	5/9 (56%) Moderate
Meneghesso, Marcatto [55] Brazil	Quasi-experimental	ACLS	6/9 (67%) Moderate
Requena-Mullor, Alarcón-Rodríguez [51] Spain	Quasi-experimental	BLS	5/9 (56%) Moderate
Roh, Kim [54] Korea	Quasi-experimental	Critical care	5/9 (56%) Moderate
Sapiano, Sammut [52] Malta	Quasi-experimental	Critical care	5/9 (56%) Moderate
Seol and Lee [49] Korea	Quasi-experimental	BLS	6/9 (67%) Moderate
Tseng, Hou [58] Taiwan	Quasi-experimental	Critical care	8/9 (88%) Good
Tucker, Urwin [56] United Kingdom	Quasi-experimental	BLS	9/9 (100%) Good
Tuzer, Inkaya [47] Turkey	Quasi-experimental	ACLS	8/9 (88%) Good
Yang and Oh [57] South Korea	Quasi-experimental	NRP	8/9 (88%) Good
Zieber and Sedgewick [27] Canada	Quasi-experimental	ACLS	5/9 (56%) Moderate

### Results of syntheses

In the following narrative analysis, findings are categorised into three themes interpreting the impact of SBL on nursing students. The first theme explores the ‘Impact of SBL on knowledge and skills acquisition,’ probing the immediate effects of SBL on the learner’s capabilities. The second theme, ‘Impact of SBL on retention of knowledge and skills,’ examines the durability of these learning outcomes over a span of time. Finally, the third theme focuses on the ‘Impact of SBL on wider clinical performance,’ which concerns the impact of SBL on broader clinical practice (self-confidence, satisfaction, clinical reasoning, self-efficacy, and problem-solving). These themes offer a comprehensive understanding of the influences of SBL in nursing education.

### Theme 1: Impact of SBL on knowledge and skills acquisition

It is evident that this emerges as the most prominent observation from this review. Despite the diversity in simulation environments, methodologies, and outcomes which encompassed a notable level of heterogeneity among the studies included, there was a steady and credible agreement suggesting that SBL is a potent method for enhancing both knowledge and skills of nursing students. This was observed in the context of CPR-based skills [26, 29, 42, 44], along with other vital skills [52, 58], and decision-making tasks [28].

Sarvan and Efe [41] utilised serious game simulation (SGS) in neonatal resuscitation training, employing a randomized controlled pre-test and post-test design. This approach led to notable improvements in practical skills, indicating the efficacy of SGS in enhancing skill acquisition. Similarly, Li et al. [40] implemented a blended learning approach integrating online virtual simulation with traditional methods in CPR training. This study observed marked improvements in self-directed learning capabilities and CPR skills, demonstrating the role of SBL in facilitating the acquisition of both cognitive and practical skills.

The effect of simulation on knowledge and skills acquisition demonstrated statistically significantly higher means for the experimental groups demonstrating a range of improvement in performance from 10 to 35% compared to the control groups in some studies. Hardenberg et al. [30] reported a mean difference of (*p* < 0.05) skills that were enhanced amongst the training simulation group. Keys et al. [32] reported that overall performance during CPR was significantly higher (*p* = 0.003) for participants in the intervention group (simulation) compared to the control group (10/10 and 5/10, respectively).

Furthermore, there was an improvement in the knowledge and skill acquisition as the review identified a consistent increase across studies, with post-intervention scores showing an increase ranging from 20 to 50% compared to pre-intervention scores. D’Cunha et al. [44] noted that there was a significant increase in mean scores in the areas of clinical reasoning, knowledge, and skills from the pre-test to the post-test (55.69–77.33%) following the simulation drills. Demirtas et al. [26] found a significant improvement in the students’ knowledge and skills of CPR following simulation training (*p* = 0.001). The mean pre-test CPR knowledge score was 5.66 ± 1.97, which increased significantly to 8.38 ± 1.30 after the simulation-based CPR training. Additionally, the mean CPR skills score improved from 22.29 ± 5.07 pre-test to 32.51 ± 1.80 post-test, resulting in a 45.9% improvement in CPR skills. indicates an effect, showing that the intervention had a significant impact on students’ learning outcomes. Kardong-Edgren et al. [50] reported a significant improvement in overall compression scores from the pre-test (M = 42.76) to the post-test (M = 77.87). This improves the effectiveness of the intervention in enhancing CPR compression performance, highlighting the critical role of SBL in improving resuscitation skills. Further, Filomeno et al. [45] observed a significant improvement in critical care scenarios such as ABCDE assessment, disturbance identification, prioritisation and application of algorithms when analysing the post-test: *p* = 0.01.

It is worth highlighting that these outcomes were consistently observed across studies with varying degrees of rigor in their design, such as Kardong-Edgren et al. [50] and Zieber and Sedgewick [27] and RCT designs such as those conducted by Kim et al. [37] and Hardenberg et al. [30]. This is crucial detail because RCT studies seek to minimise the impact that confounding variables have on the results of intervention studies and are hence considered a more robust paradigm [64]. The range of RCT studies incorporated within this review supply evidence that consistently supports the findings of the non-randomised studies and therefore enables a greater degree of confidence to be assigned to this theme. The consistently positive findings throughout the selected SBL studies result in the conclusion that this method is an effective means of boosting the skills and knowledge of nursing students.

### Theme 2: Impact of SBL on retention of knowledge and skills

The second theme, the impact of SBL on long-term retention of clinical knowledge and skills, extends the findings of the first theme by examining the sustainability of the acquired knowledge and skills beyond the initial SBL session. The first theme arose from consistent findings across varied settings, outcomes, and designs, while the second theme lacks universality due to limited comprehensive analyses in the *n* = 33 studies concerning meaningful long-term retention, and due to mixed results. Within the theme of retention, eight studies were assessed the retention. Six of these studies reported 15% improvements, while two observed a 5% decline following the intervention. Notably, the follow-up period across these studies varied, with a range of two to five months. This variation provides insights into the short-term impacts of the interventions on retention rates. For instance, Charlier et al. [43] through a quasi-experimental study, demonstrated sustained retention of Basic Life Support (BLS) skills and knowledge at four-month follow-up among participants who underwent SBL. Similar results were reported by Arrogante et al. [29], who found superior CPR performance in the SBL group compared to controls after three months. Further, RCTs by Padilha et al. [35] and Araújo et al. [34] also supported the hypothesis that SBL fosters improved cognitive retention and performance over traditional methods. Habibli et al. [31] observed enhanced nursing students’ BLS-CPR knowledge and performance due to SBL. At three-month follow-up, the intervention group scored higher (15.07, 16.57) compared to the control group (13.33, 14.76). Zieber and Sedgewick [27] corroborated these findings.

However, Seol and Lee [49] and Tuzer et al. [47] did not find retention of knowledge and skills acquired through SBL at 20-week follow-up. Though these findings contradict others, it is important to consider that Seol and Lee’s study had a small sample size (*n* = 32), potentially affecting its validity and reliability. These discrepancies suggest a need for further research on the long-term retention of knowledge and skills through SBL.

### Theme 3: Impact of SBL on wider clinical performance

SBL’s extended impact encompasses skills and knowledge and broader clinical performance, including enhancements in self-confidence, satisfaction, clinical reasoning, self-efficacy, and problem-solving. Tucker et al. [56] investigated the effect of simulated scenarios on nursing students’ self-efficacy in resuscitation and found that such simulations bolster student confidence, a crucial component of clinical performance. Moreover, the study by Meneghesso et al. [55]using the ‘blindfolded’ technique in clinical simulations highlighted an increase in self-confidence and knowledge, which are vital for effective clinical performance. Additionally, the research by Li et al. [40] and Yang and Oh [57] demonstrated improvements in problem-solving abilities and learning motivation, underscoring the positive influence of SBL on various facets of clinical performance. These improvements were highlighted in RCTs conducted by Padilha et al. [35], Kim et al. [37], Svellingen et al. [28], Seo and Eom [39], Tawalbeh [38], and non-randomized studies by Charlier et al. [43], Filomeno et al. [45], Demirtas et al. [26], Goldsworthy et al. [46], and Lau et al. [53]. Additionally, studies by Roh et al. [54] and Lau et al. [53] indicated that SBL is correlated with improved teamwork and collaboration, crucial elements in clinical practice [65]. These findings represent insightful benefits of SBL and suggest an area that merits further exploration in future research.

### Risk of bias across studies

The studies reported demographic information, but no significant differences concerning age, education level, or gender were detected. All RCTs employed randomization to counter selection bias, which not posed concern of possible bias.

Only five studies adopted blind designs: Keys et al. [32], Sarvan and Efe [41], Ka Ling et al. [33], Arrogante et al. [28], and Hardenberg et al. [29]. Blind design, wherein neither participants nor experimenters know group allocations, minimizes bias and bolsters result validity [19]. Ka Ling et al. [32] and Sarvan and Efe [41] utilised a double-blind approach, keeping both participants and assessors unaware of study aims and group memberships, curbing assessment biases. Conversely, Keys et al. [31], Arrogante et al. [28], and Hardenberg et al. [29] applied single-blind designs for course deliverers and assessors, minimizing biases in course delivery and learning outcome assessments.

Implementing blind design in simulation studies can be complex due to the simulation environment’s nature, where experimenters might access information revealing group assignments, and ensuring participant blinding may be challenging. However, careful planning can facilitate blind design integration in simulation studies.

## Discussion

This review aimed to systematically evaluate the literature to determine the effectiveness of SBL in knowledge and skills acquisition and retention. While certain topics, such as CPR skills or specific simulation interventions, could potentially be for meta-analysis, the overall heterogeneity of the included studies, particularly in terms of intervention contexts, outcome measures, and reporting, limited the feasibility of conducting a meta-analysis across all studies.

Evidence from 33 primary research studies indicates a positive association between SBL and improvements in knowledge and skills. The majority of these studies focused on skills related to CPR and basic life support [26, 45]. Other studies examined different clinical skills, such as critical care skills [30, 46] and clinical decision-making [28]. As noted previously, simulations facilitate practice of emergency response procedures and specialised equipment operation that may not be sufficiently encountered through standard clinical placements [5]. Performing high-fidelity CPR and managing dynamic patient deterioration events further require sophisticated clinical judgement and psychomotor proficiencies that simulation-based mastery learning allows novice nurses to acquire [4].

While SBL demonstrated enhanced clinical skills and knowledge acquisition within the included studies, evidence supporting retention remains preliminary and constrained by limited longitudinally. Only 8 of the 33 included studies measured outcome durability over time, with retention follow-up assessments spanning just two to five months post-intervention. However, since this time period is narrowly constrained, it does not provide adequate opportunity for long-term knowledge and skill maintenance [49].

Furthermore, certain studies revealed additional benefits of SBL. For instance, Demirtas et al. [26], Tawalbeh [38], and Goldsworthy et al. [46] reported enhancements in learners’ confidence and self-efficacy, which might lead to improved clinical performance and patient outcomes. Roh et al. [54] and Lau et al. [53] indicated that SBL sessions were linked with enhanced teamwork and collaboration, which are vital in clinical practice [65]. These results imply that the impact of SBL may extend beyond knowledge and skills acquisition and retention, meriting further investigations to understand the underlying mechanisms in various healthcare settings.

While the current systematic review aligns with prior evidence syntheses in finding SBL effective for developing nursing knowledge and skills, key differences in scope and methodology underpin its unique contributions. Al Gharibi and Arulappan’s [11] review of 11 studies focused specifically on improved confidence and competence regarding clinical skills between 2011 and 2019. Alternatively, this review captured a rapidly expanding literature base of over 30 studies through near-current 2023 searches. This enabled more comprehensive evaluation across multidimensional impacts including knowledge acquisition, psychomotor skill development, clinical judgement and long-term retention, with 16 randomized controlled trials denoting higher quality evidence.

Additionally, Labrague et al.’s descriptive review examined simulation’s effect on anxiety but was restricted to correlational inquiries rather than experimental research evaluating intra-individual skill and knowledge growth. Furthermore, neither of these prior reviews substantially addressed sustainability questions regarding simulation training’s enduring effects on retention over extended periods. Thus, the current systematic review significantly builds upon preceding evidence by consolidating demonstrable knowledge and skill-based effectiveness data across a substantial set of controlled interventions. However, it only identified eight longitudinal studies analysing retention outcomes across a two to five-month timeframe. Therefore, the review highlights long-term retention as a critical yet understudied domain warranting markedly expanded ongoing investigation through longitudinal inquiries to firmly determine the sustainability of simulation training impacts.

Although some studies addressed fidelity variations, only Tuzer et al. [47] compared fidelity forms and did not find conclusive evidence for the superiority of one approach over another. Massoth et al. [10] had similar findings but reported overconfidence in high-fidelity SBL participants, a factor not examined in the present review.

Regarding study quality, the inclusion of 16 RCTs lends credibility to the review, but it is noteworthy that an equal number of studies did not employ randomization, which may impact the quality of evidence [64]. Additionally, large sample sizes in some non-randomized trials like Requena-Mullor et al. [51] could offset limitations by providing statistical power [66]. Conversely, some RCTs had small sample sizes like Hardenberg et al. [30], raising concerns about statistical power and reliability [67]. This emphasizes the need for rigorous evaluation during the research design phase to ensure both scientific and ethical integrity [68].

### Strengths and limitations

This systematic review adopted a rigorous approach aligned with best practice standards, as reinforced through its registration with PROSPERO and adherence to the new PRISMA guidance. Comprehensive searches of major databases identified relevant literature without geographical constraints, facilitating the inclusion of 33 recent experimental studies from 2017 to 2023. This selective date range allowed targeted insight into simulation pedagogy maturation. Additionally, the review exclusively synthesized quantitative experimental research across 15 randomized controlled trials and 18 quasi-experimental designs to optimize internal validity in assessing simulation effectiveness. Paired screening and duplicate data extraction further minimized subjectivity and bias.

This systematic review sheds light on SBL within nursing education but is subject to limitations in both the included studies and the review process. The evidence included is limited by variability in study quality, with the absence of blinding, and small sample sizes, potentially undermining reliability and generalizability. Additionally, the heterogeneity in SBL intervention characteristics, such as duration, intensity, and design, renders comparison challenging. Transitioning to the review methodology, its scope is curtailed by an exclusion of non-English language, introducing potential language biases. Furthermore, the review’s narrow focus on SBL and its temporal constraint to recent publications may bypass valuable past findings.

### Implications for nursing education and future research

Despite demonstrating effectiveness for knowledge and skill acquisition, constraints remain with regard to enduring retention. With few studies assessing beyond five months and an absence of longitudinal studies, the long-term sustainability of learning benefits remains inadequately elucidated. Given substantial knowledge gaps regarding the long-term impact of simulation, nurse educators should hold realistic expectations for knowledge and skills retention when incorporating simulation methodologically. To maximize efficacy, it is imperative that educators integrate a diversity of SBL modalities, such as high-fidelity simulation and role-playing, tailored to distinct learning needs and curricular objectives.

Academic institutions, including universities and nursing colleges, must foster collaboration with nursing educators and be committed to the integration of SBL into curricula. This necessitates investments in faculty training, simulation equipment, and technology. It is incumbent upon these institutions to ensure that curricular integration is resource-supported, aligning with educational objectives and addressing the distinctive learning requisites of nursing students.

Future studies should scrutinize the optimal utilisation of SBL, including efficacious teaching methodologies, the correlation between SBL duration and knowledge retention, and the transferability of knowledge and skills to clinical contexts. Longitudinal studies could elucidate the long-term implications of SBL on students’ competency and the impact on diverse cohorts. Moreover, the adoption of SBL necessitates significant resource investments in equipment, facilities, technologies, and specialized staff. Without demonstrating a favourable return on investment, the simulation could be priced out of reach. Therefore, an examination of cost-effectiveness regarding traditional methods, and more analysis of barriers and facilitators to SBL implementation would be invaluable in optimizing the quality of nursing education and preparing adept nursing professionals for the dynamic healthcare landscape.

## Conclusions

This systematic review suggests that SBL is an effective pedagogical approach for promoting knowledge and skill acquisition and retention across a range of nursing education topics, including cardiopulmonary and critical care among nursing students. However, evidence gaps persist regarding enduring skill and knowledge retention outcomes.

With fewer than 25% of included studies assessing retention beyond five months post-intervention, current findings lack generalizability concerning the long-term sustainability of simulation’s learning impacts. Furthermore, this review provides a set of findings which both support and extend previous work in this area. The analysis of both randomized controlled and quasi-experimental studies demonstrated consistent and significant improvements in various measures of learning outcomes, including knowledge, skills, and self-confidence. These findings are particularly relevant given the increasing demand for nursing education programmes to prepare students for the complexities and challenges they will face in contemporary healthcare environments. Nonetheless, the evidence is tempered by limitations including heterogeneity in study designs and risk of bias. These constraints highlight the imperative for rigorous research to clarify the optimal parameters for SBL deployment and to investigate its applicability to diverse cohorts and clinical environments. Overall, this systematic review lends qualified support for simulation-based learning as a potentially valuable experiential teaching strategy within nursing education, though efficacy conclusions must be interpreted cautiously given considerable evidence gaps, particularly regarding enduring knowledge and skill retention impacts.

## Supplementary Information

Supplementary Material 1: Results of critical appraisal risk of bias for Randomised Controlled Trials studies.

Supplementary Material 2: Results of critical appraisal and risk of bias for quasi-experimental design studies.

## Data Availability

The data that supports the results and findings of this systematic review can be found in either the main paper or the additional supporting files. Any other data from the current study are available from the corresponding author upon reasonable request.
