# Supplementary material for: The effectiveness of simulation-based learning (SBL) on students’ knowledge and skills in nursing programs: a systematic review
Source: BMC Med Educ. 2024 Oct 7;24:1099. doi: 10.1186/s12909-024-06080-z (PMC11459713; doi:10.1186/s12909-024-06080-z)
Supplement: Supplementary file 1 — Supplementary Material 1: Results of critical appraisal risk of bias for Randomised Controlled Trials studies. [file 12909_2024_6080_MOESM1_ESM.docx]

**Appendix A**

**Results of critical appraisal for Randomised Controlled Trials**

| **JBI checklist criteria (potential bias)** | | | | | | | | | | | | | | |
| --- | --- | --- | --- | --- | --- | --- | --- | --- | --- | --- | --- | --- | --- | --- |
| **Main author** | **1** | **2** | **3** | **4** | **5** | **6** | **7** | **8** | **9** | **10** | **11** | **12** | **13** | **Total (%) and quality rating*** |
| **Araújo, Medeiros (34)** | Y | U | Y | U | U | U | Y | Y | Y | Y | Y | Y | Y | 9/13 (69%) Moderate |
| **Arrogante, Rios-Diaz (29)** | Y | N | Y | N | Y | Y | Y | Y | Y | Y | Y | Y | U | 10/13 (77%) Moderate |
| **Farsi, Yazdani (42)** | Y | Y | Y | N | N | U | Y | Y | Y | Y | Y | Y | U | 9/13 (69%) Moderate |
| **Habibli, Ghezeljeh (31)** | Y | N | Y | U | U | U | Y | Y | Y | Y | Y | Y | U | 8/13 (62%) Moderate |
| **Hardenberg, Rana (30)** | Y | U | Y | U | Y | Y | Y | Y | Y | Y | Y | Y | U | 10/13 (77%) Moderate |
| **Ka Ling, Lim Binti Abdullah (33)** | Y | U | Y | Y | U | Y | Y | Y | Y | Y | Y | Y | Y | 11/13 (85%) Good |
| **Keys, Luctkar-Flude (32)** | Y | N | Y | N | Y | Y | Y | Y | Y | Y | Y | Y | N | 10/13 (77%) Moderate |
| **Kim, Issenberg (37)** | Y | N | Y | U | U | N | Y | Y | Y | Y | Y | Y | N | 8/13 (62%) Moderate |
| **Li, Lv (40)** | Y | Y | Y | U | U | U | Y | Y | Y | Y | Y | Y | Y | 10/13 (77%) Moderate |
| **Padilha, Machado (35)** | Y | Y | Y | U | U | U | Y | Y | Y | Y | Y | Y | Y | 10/13 (77%) Moderate |
| **Saeidi and Gholami (36)** | U | N | Y | U | U | U | Y | Y | Y | Y | Y | Y | N | 7/13 (54%) Moderate |
| **Sarvan and Efe (41)** | Y | Y | Y | Y | N | Y | Y | Y | Y | Y | Y | Y | Y | 11/13 (85%) Good |
| **Seo and Eom (39)** | Y | U | Y | N | N | N | Y | Y | Y | Y | Y | Y | Y | 9/13 (69%) Moderate |
| **Svellingen, Forstrønen (28)** | Y | N | Y | N | N | N | Y | Y | Y | Y | Y | Y | U | 8/13 (62%) Moderate |
| **Tawalbeh (38)** | Y | Y | Y | U | N | U | Y | Y | Y | Y | Y | Y | Y | 10/13 (77%) Moderate |
| **1.** Was true randomization used for assignment of participants to treatment groups? (selection bias) 2**.** Was allocation to treatment groups concealed? (selection bias) **3.** Were treatment groups similar at the baseline? (selection bias/design bias) **4.** Were participants blind to treatment assignment? (performance bias) **5.** Were those delivering treatment blind to treatment assignment? (performance/detection bias) **6.** Were outcomes assessors blind to treatment assignment? (ascertainment bias) **7.** Were treatments groups treated identically other than the intervention of interest? (systematic difference/containment bias) **8.** Was follow-up complete, and if not, were strategies to address incomplete follow-up utilized? (attrition bias) **9.** Were participants analysed in the groups to which they were randomized? (intention to analysis) **10.** Were outcomes measured in the same way for treatment groups? (instrumentation/testing effects threats) **11.** Were outcomes measured in a reliable way? (measurement bias) **12.** Was appropriate statistical analysis used? (performance/detection bias) **13.** Was the trial design appropriate, and any deviations from the standard RCT design (individual randomization, parallel groups) accounted for in the conduct and analysis of the trial? (design bias) | | | | | | | | | | | | | | |

***Good: at least 80%; Moderate: 50–80%; Poor: less than 50%**

**Key: Yes =Y No= N Unclear = U** **Not applicable = NA**
