# Supplementary material for: The effectiveness of simulation-based learning (SBL) on students’ knowledge and skills in nursing programs: a systematic review
Source: BMC Med Educ. 2024 Oct 7;24:1099. doi: 10.1186/s12909-024-06080-z (PMC11459713; doi:10.1186/s12909-024-06080-z)
Supplement: Supplementary file 2 — Supplementary Material 2: Results of critical appraisal and risk of bias for quasi-experimental design studies. [file 12909_2024_6080_MOESM2_ESM.docx]

**Appendix B**

**Results of critical appraisal for quasi-experimental studies**

| **JBI checklist criteria (potential bias)** | | | | | | | | | | |
| --- | --- | --- | --- | --- | --- | --- | --- | --- | --- | --- |
| **Main author** | **1** | **2** | **3** | **4** | **5** | **6** | **7** | **8** | **9** | **Total (%) and quality rating*** |
| **Charlier, Van Der Stock (43)** | Y | Y | Y | N | Y | Y | Y | Y | Y | 8/9 (88%) Good |
| **Chen, Yang (48)** | Y | N | Y | Y | Y | Y | Y | Y | Y | 8/9 (88%) Good |
| **D’Cunha, Fernandes (44)** | Y | Y | Y | N | Y | Y | Y | Y | Y | 8/9 (88%) Good |
| **Demirtas, Guvenc (26)** | Y | Y | Y | N | Y | Y | Y | Y | Y | 8/9 (88%) Good |
| **Filomeno, Renzi (45)** | Y | Y | Y | N | Y | N | Y | Y | Y | 7/9 (78%) Moderate |
| **Goldsworthy, Patterson (46)** | Y | Y | N | Y | Y | Y | Y | Y | Y | 8/9 (88%) Good |
| **Kardong-Edgren, Oermann (50)** | Y | NA | NA | N | Y | Y | NA | Y | Y | 5/9 (56%) Moderate |
| **Lau, Chee (53)** | Y | N | NA | N | Y | Y | NA | Y | Y | 5/9 (56%) Moderate |
| **Meneghesso, Marcatto (55)** | Y | NA | NA | N | Y | Y | Y | Y | Y | 6/9 (67%) Moderate |
| **Requena-Mullor, Alarcón-Rodríguez (51)** | Y | NA | NA | N | Y | Y | NA | Y | Y | 5/9 (56%) Moderate |
| **Roh, Kim (54)** | Y | NA | NA | N | Y | Y | NA | Y | Y | 5/9 (56%) Moderate |
| **Sapiano, Sammut (52)** | Y | NA | NA | N | Y | Y | NA | Y | Y | 5/9 (56%) Moderate |
| **Seol and Lee (49)** | Y | NA | NA | N | Y | Y | Y | Y | Y | 6/9 (67%) Moderate |
| **Tseng, Hou (57)** | Y | Y | Y | Y | Y | Y | Y | Y | Y | 8/9 (88%) Good |
| **Tucker, Urwin (56)** | Y | Y | Y | Y | Y | Y | Y | Y | Y | 9/9 (100%) Good |
| **Tuzer, Inkaya (47)** | Y | Y | Y | N | Y | Y | Y | Y | Y | 8/9 (88%) Good |
| **Yang and Oh (57)** | Y | Y | N | Y | Y | Y | Y | Y | Y | 8/9 (88%) Good |
| **Zieber and Sedgewick (27)** | Y | NA | NA | N | Y | Y | NA | Y | Y | 5/9 (56%) Moderate |
| **1.** Is it clear in the study what is the ‘cause’ and what is the ‘effect’ (i.e. there is no confusion about which variable comes first)? (causation/reverse causation) **2.** Were the participants included in any comparisons similar? (selection bias) **3.** Were the participants included in any comparisons receiving similar treatment/care, other than the exposure or intervention of interest? (history threat/systematic difference/ contamination bias) **4.** Was there a control group? (measurement bias) **5.** Were there multiple measurements of the outcome both pre and post the intervention/exposure? (maturation threat, regression to the mean) **6.** Was follow-up complete, and if not, was follow-up adequately reported and strategies to deal with loss to follow-up employed? (attrition bias) **7.** Were the outcomes of participants included in any comparisons measured in the same way? (instrumentation/testing effects threats) **8.** Were outcomes measured in a reliable way? (detection/instrument/ measurement bias) **9.** Was appropriate statistical analysis used? (performance/detection bias) | | | | | | | | | | |

***Good: at least 80%; Moderate: 50–80%; Poor: less than 50%**

**Key: Yes =Y No= N** **Unclear= U Not applicable = NA**
